# Next Generation and Other Sequencing Technologies in Diagnostic Microbiology and Infectious Diseases

**DOI:** 10.3390/genes13091566

**Published:** 2022-08-31

**Authors:** Evann E. Hilt, Patricia Ferrieri

**Affiliations:** Department of Laboratory Medicine and Pathology, University of Minnesota, Minneapolis, MN 55455, USA

**Keywords:** next-generation sequencing, microbiology, diagnostics

## Abstract

Next-generation sequencing (NGS) technologies have become increasingly available for use in the clinical microbiology diagnostic environment. There are three main applications of these technologies in the clinical microbiology laboratory: whole genome sequencing (WGS), targeted metagenomics sequencing and shotgun metagenomics sequencing. These applications are being utilized for initial identification of pathogenic organisms, the detection of antimicrobial resistance mechanisms and for epidemiologic tracking of organisms within and outside hospital systems. In this review, we analyze these three applications and provide a comprehensive summary of how these applications are currently being used in public health, basic research, and clinical microbiology laboratory environments. In the public health arena, WGS is being used to identify and epidemiologically track food borne outbreaks and disease surveillance. In clinical hospital systems, WGS is used to identify multi-drug-resistant nosocomial infections and track the transmission of these organisms. In addition, we examine how metagenomics sequencing approaches (targeted and shotgun) are being used to circumvent the traditional and biased microbiology culture methods to identify potential pathogens directly from specimens. We also expand on the important factors to consider when implementing these technologies, and what is possible for these technologies in infectious disease diagnosis in the next 5 years.

## 1. Introduction

The use of next-generation sequencing (NGS) technologies in clinical diagnostics has increased over the past several decades. NGS technologies allow for high throughput, massively parallel sequencing of millions of fragments of DNA. In the realm of genetic disorders and cancer diagnostics, these technologies have become the diagnostic gold standard. It was not until the last decade, when these NGS technologies became more readily available, that the use of these technologies in the clinical microbiology laboratory has become a reality.

Current NGS technologies can be utilized in three different applications in the clinical microbiology laboratory: whole genome sequencing (WGS), targeted metagenomics sequencing and shotgun metagenomics sequencing. In this review, we analyze these three applications and provide a comprehensive summary of how these applications are currently being used in the public health, basic research, and clinical microbiology laboratory environments. These applications cannot easily be introduced into the laboratory, so we stress the important factors to consider when implementing these technologies. In addition, these technologies have already changed the landscape of the conventional clinical microbiology laboratory, and we discuss what we believe is possible for these technologies in infectious disease diagnostics in the next 5 years.

## 2. Evolution of Sequencing Technologies

Sequencing technologies have rapidly grown in the last 60 years, starting with first generation sequencing and evolving into the current third generation sequencing technologies [1] (Table 1). The genesis of first generation sequencing occurred simultaneously when both Frederick Sanger and the joint pair of Allan Maxam and Walter Gilbert released their protocols of how to sequence DNA [2,3]. The Maxam-Gilbert method of sequencing was based on chemical fracture where radiolabeled DNA is treated with various chemicals to break the chain at specific bases. These fragments were then run on a polyacrylamide gel to determine the position of the specific nucleotide of interest [3]. Sanger proposed the chain-termination method that involves the use of chemical analogs to deoxynucleotides (dNTPs), known as dideoxynucleotides (ddNTPs), which prevent further extension of the DNA chain. The method involves four parallel reactions with ddNTPs that are run along a polyacrylamide gel to interpret which base is present in the nucleotide sequence [2]. It was improvements made to the Sanger’s chain-termination method, such as changing to fluorometric based detection [4,5] and detection with capillary based electrophoresis [6,7], that advanced the sequencing of DNA to automated DNA sequencing instruments [8].

The second generation of sequencing technologies emerged when, instead of radio-labeled or fluorescently labeled nucleotides, researchers measured pyrophosphate synthesis with luminescence to determine the nucleotide sequence [9,10,11,12]. This method known as “pyrosequencing” and the instruments built to perform pyrosequencing allowed for a massive parallel of sequence reactions to take place, which increased the amount of DNA that can be sequenced in a single run [13]. Other parallel sequencing techniques evolved, including sequencing by oligonucleotide ligation and detection (SOLiD) system [Applied Biosystems (Waltham, MA, USA)], which sequenced DNA by ligation [14,15] and the Ion Torrent method of sequencing, which measures the difference in pH caused by release of protons during polymerization [16]. The most notable and successful of these second generation sequencing techniques is the bridge amplification method of sequencing done initially by Solexa, which was later acquired by Illumina (San Diego, CA, USA) [17,18]. This method involves adapter labelled fragmented DNA passing over a lawn of complimentary oligonucleotides bound to a flow cell. Once bound, a solid phase polymerase chain reaction (PCR) produces clusters of clonal populations from each individual original flow-cell binding DNA strand [18]. The first of these Illumina Genomic Analyzer instruments was only able to produce very short reads, but it generated paired-end data with a sequence at each end [19]. These paired-end data provide a larger amount of information that offers greater accuracy with less background noise compared to other second generation techniques [20,21,22].

There is still no agreement on what defines the difference between second and third generation sequencing technologies, but for the purpose of this review, we define third generation sequencing as the ability to perform single molecule sequencing. Single molecule sequencing technologies do not require DNA amplification steps; therefore, allowing for higher throughput, faster turnaround times and longer read lengths being produced [23,24]. Several companies have led the way in these third generation sequencing technologies and the two most notable are Pacific Biosciences (PacBio; Menlo Park, CA, USA) and Oxford Nanopore Technologies (Oxford, UK). The PacBio method measures DNA polymerase incorporation of fluorescently labeled nucleotides onto a complementary sequence template. At the center of this technology is a dense array of zero-mode wavelength (ZMW) nanostructures [25]. These ZMW nanostructures allow for measurement of the individual fluorescently labeled nucleotides to take place in real-time and a short amount of time. This technology is capable of producing very long reads up to 10 kilobases (kb) in length [25]. The Oxford Nanopore Technologies method uses nanopores, both biological and solid-state, embedded in a membrane with an ionic current [26]. Single-stranded genomic DNA or RNA can pass through the nanopores, and each individual nucleotide base physically blocks the current, which can be measured with standard electrophysiological techniques [27].

**Table 1:** **Comparison** **of Methods across Sequencing Generations**.

	Method	Technology ^a^	Throughput (Low or High)	Complexity (Moderate or High)	Use for Sequencing in Relation to Clinical Microbiology	References
**First Generation**	Maxam-Gilbert	Chemical fracture of radiolabeled DNA at specific bases	Low	Moderate	N/A	[3]
	Sanger	Chain-termination at specific bases using dideoxynucleotides	Low	Moderate	16S and 28S Identification Whole Genome Sequencing	[2,4,5,6,7,8]
**Second Generation**	Pyrosequencing	Measure of pyrophosphate synthesis with luminescence	High	High	Whole Genome Sequencing	[9,10,11,12,13]
	SOLiD	Measure of DNA ligation of oligonucleotide	High	High	Whole Genome Sequencing	[14,15]
	Ion Torrent	Measurement of pH difference in release of protons during polymerization of DNA	High	High	Whole Genome Sequencing	[16]
	Illumina	Bridge amplification method	High	High	Whole Genome Sequencing Deep Amplicon Sequencing Shotgun Metagenomics	[17,18,19]
**Third Generation**	PacBio	Single-molecule resolution using zero-mode wavelength (ZMW) nanostructures	Moderate	High	Whole Genome Sequencing	[25]
	Nanopore	Single-molecule resolution using biological and solid-state nanopores	Moderate	High	Whole Genome Sequencing Deep Amplicon Sequencing Shotgun Metagenomics	[26,27]

^a^ See text section “Evolution of Sequencing Technologies” for more detailed description of the technologies.

In relation to current clinical microbiology diagnostics, first generation Sanger sequencing technology has been utilized to identify both bacterial and fungal species from direct colony growth [28,29,30,31,32]. For bacterial species, the 16S ribosomal RNA (rRNA) and DNA-dependent RNA polymerase β-subunit (*rpoB*) genes are used for identification [29,33], while for fungal species, the ribosomal internal transcribed spacer (ITS) and 28S rRNA genes are used for identification [34,35,36]. The newer generations of sequencing (second and third) are currently being explored for use in the clinical microbiology diagnostic setting to identify pathogenic organisms of interest. There are two major ways in which this is being accomplished; the first is an enhancement of identifying what is growing in culture through whole genome sequencing (WGS). The second is the use of metagenomics sequencing to identify potential pathogenic organisms directly from the source to bypass the culture process and/or to enhance the outcome. Both avenues will be discussed for the remainder of the review.

## 3. Whole Genome Sequencing of Microorganisms

Whole genome sequencing (WGS) is the process of sequencing and assembling the microbial genome of an organism of interest. These microbial genomes can represent bacteria, fungi, and viral organisms. WGS for bacteria, mycobacteria and fungal organisms requires culture and isolation of the organism prior to the nucleic acid extraction and the subsequent sequence pipeline. This is a limitation for organisms that are difficult to grow or unable to grow in culture. In the case of viral genomes, WGS is utilized by sequencing the sample directly for the viral genome of interest and will be discussed later in the metagenomics sequencing section.

**Figure 1:**
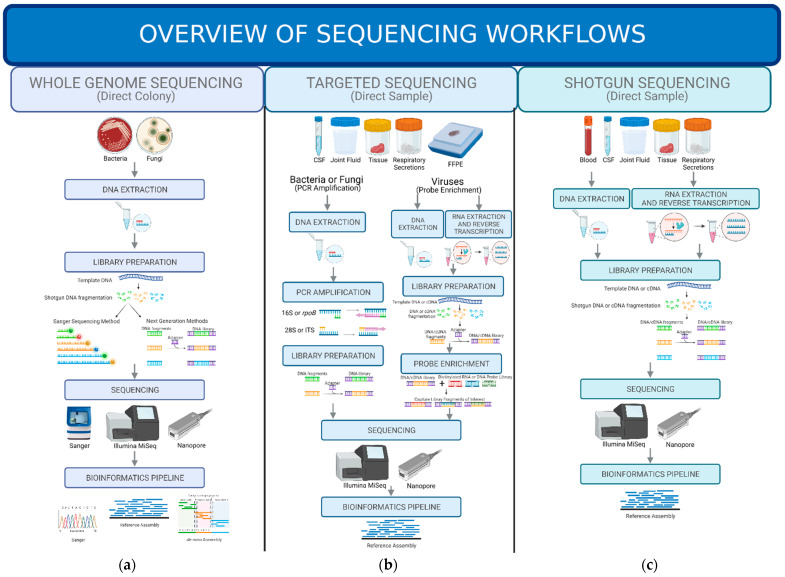
**Overview of Sequencing Workflows.** (**a**) Whole Genome Sequencing-This workflow begins with a colony from a microorganism of interest. Next, DNA is extracted and then fragmented and placed through a library preparation for either Sanger Sequencing or other NGS methods. The library is then sequenced and analyzed with a bioinformatics pipeline. (**b**) Targeted Sequencing- This workflow is one that begins with the clinical sample and involves a selection or enrichment process prior to library preparation in the case of bacteria and fungi. If the pathogen of interest is a virus, the selection or enrichment occurs after the library preparation. The library prepped samples are then sequenced and analyzed with a bioinformatics pipeline. (**c**) Shogun Sequencing-This workflow is similar to the workflow of WGS but instead of a colony of the microorganism of interest, the DNA or RNA is extracted directly from the clinical sample submitted. This extracted DNA or RNA is then placed through a library preparation and then sequenced. The results are analyzed with a bioinformatics pipeline. Figure created with BioRender.com (accessed on 24 August 2022).

The NGS technologies used for WGS are either, second generation sequencing (e.g., Illumina) or third generation sequencing technology (e.g., PacBio or Nanopore), and the advantages and disadvantages of using either are described in more detail elsewhere [37]. There are many details for how one takes a pure culture of an organism from the culture plate to the final sequencing results with both generation technologies, but the overall workflow is the same (Figure 1). In brief, the organism is first removed from the plate and the DNA is extracted. Once the DNA is extracted, a library is created where each individual organism’s DNA is sheared into fragments and given adapters that contain a unique bar code to enable multiplexing of hundreds of samples. These individual libraries are pooled together and submitted to the NGS technology of choice. Once sequencing has completed, bioinformatics is performed to de-multiplex the samples and then quality filtering and adapter removal are performed. Then, there are three ways to assembly the genome to obtain an identification with WGS [38]. The first is known as reference assembly where the DNA fragments are aligned to a known reference genome and a consensus genome is obtained, like a puzzle. The second is de novo assembly where all the DNA fragments are assembled into contigs. With de novo assembly, it is difficult to obtain high quality genomes [39], which is why a more popular third assembly pipeline is a hybrid of de novo and reference assemblies, where the DNA fragments are assembled into contigs and then the contigs are then mapped to reference genomes.

WGS on microorganisms that can be cultured and isolated can be applied to aid in identification of the organism, typing of the organism for epidemiologic purposes and detection of possible antimicrobial resistance of the organism [40]. Conventional clinical microbiology methods for the initial steps of identification of cultured bacteria include basic morphological observations, biochemical tests, and identification with matrix-assisted laser desorption-ionization time-of-flight mass spectrometry (MALDI-TOF MS); the latter is still very accurate and fast compared to WGS [40]. However, there are instances when MALDI-TOF MS cannot make a confident identification of the species identification especially with fastidious organisms [41] and anaerobic bacteria [42]. In most instances, identification to the genus level is sufficient, but there are some instances, where species identification is imperative due to antibiotic susceptibility profile differences between species. For example, *Enterococcus faecium* versus *Enterococcus faecalis* have two different antibiotic susceptibility profiles that are clinically and epidemiologically relevant [43]. *E. faecium* is intrinsically resistant to ampicillin, and this species of *Enterococcus* has a high rate of vancomycin resistance, where both are relatively rare to find in *E. faecalis* [43]. Now MALDI-TOF MS has a very accurate identification between these two species of *Enterococcus*, but this is an extreme example to show that genus identification is not always sufficient.

Current microbiology identification methods are unable to identify the serotype of a bacterium, which is important in cases of *Salmonella* infection where it is critical to identify enteric fever for treatment [44]. Here, in the United States, serotype identification of *Salmonella* by WGS is currently being performed at public health laboratories. Prior to the use of WGS, public health laboratories used pulse-field gel electrophoresis (PFGE) for *Salmonella* serotype identifications [45], but comparison studies of WGS demonstrated that it was comparable to PFGE and it has become the gold standard reference method [46,47,48]. There are many different bioinformatics approaches that are used with WGS to type a particular isolate and one of the main approaches is known as multi-locus sequence typing (MLST). This approach uses a set of “house-keeping” genes (5–7 genes depending on the bacterium) for a particular bacterial species and uses mutations within those genes to compare how related one bacterial isolate is to another [49]. MLST, with designation of sequence types (STs), has been long been the gold-standard for typing of organisms using WGS analysis. However, Leekitcharoenphon et al. 2014 investigated several types of bioinformatics analyses to compare various WGS typing methods to PFGE for outbreak clustering [47]. They compared 18 isolates of *Salmonella enterica* serovar Typhimurium from six different outbreaks with PFGE and four different bioinformatics typing approaches and found that single nucleotide polymorphism (SNP) analysis was the approach that was able to cluster the 18 isolates into their respective outbreak clusters with 100% concordance [47]. Although not the gold standard, more laboratories are favoring the SNP analysis for epidemiologic tracking of potential outbreaks as this analysis considers the entire genome and not just a few select genes.

In the United States, the WGS results generated by public health laboratories are curated and maintained by PulseNet, which is a network of public health and food regulatory agency laboratories coordinated by the Centers for Disease Control (CDC). This network’s main task is to help identify and investigate potential outbreaks in the food production and distribution systems, which is a major initiative of the CDC approach known as One Health [50]. In addition to *Salmonella* serotype identification mentioned above, PulseNet also performs WGS to subtype and identify the following microorganisms: *Escherichia coli* (O157 and other *Shiga* toxin-producing *E. coli*), *Campylobacter*, *Listeria monocytogenes*, *Shigella*, *Vibrio cholerae*, *Vibrio parahaemolyticus* and *Cronobacter* [51].

The serotype identifications of foodborne illness and disease outbreaks are not the only areas in the public health space where WGS is utilized. WGS is also used in surveillance of vaccine-preventable diseases such as *Neisseria meningitidis* serogroups, *Streptococcus pneumoniae* serotypes [52] and antimicrobial resistant pathogens such as multi-drug resistant *Mycobacterium tuberculosis* [53,54]. One vaccine-preventable disease requiring constant monitoring is *N. meningitidis* since there are numerous outbreaks occurring annually [55]. The ability to determine the serogroup and perform WGS of *N. meningitidis* isolates are key to aid in the public health response to determine if an outbreak is occurring and if public health interventions such as mass vaccination are needed [56].

A basic research application of WGS was to characterize serotype IV group B *Streptococcus* (GBS) isolates from invasive disease in neonates that emerged in the United States and Canada [57,58]. There are diverse genetic backgrounds among serotype IV GBS isolates, including several different STs and clonal complexes (CC) [57]. Of interest, is the highly prevalent ST-452 lineage, assigned to clonal complex CC23, described from several countries [59,60]. WGS and phylogenetic analysis of the core genome suggested that ST-452 could have originated through genetic recombination with the original event and founder strain emanating from a single gene transfer between CC23 and the hypervirulent CC17 lineage [60]. Chromosomal mapping of major GBS virulence factors revealed that ST-452 strains have a unique profile among both CC23 and CC17 strains. Conjugation and homologous recombination with exchange of large chromosomal fragments, spanning hundreds of kilobases, is considered one of the major events controlling the continuing evolution of GBS [60]. Antimicrobial resistance in GBS has also been studied by WGS with examination of large numbers of GBS colonizing isolates from pregnant women [61]. The majority of serotype IV GBS isolates in this study were ST-459, a tetracycline, erythromycin, and clindamycin resistant ST, first described from Minnesota, USA [59], which is considered to be the main driver of serotype IV GBS in North America [61]. WGS studies of recombinatorial events revealed multiple episodes of capsular switching among these isolates [61]. The striking genetic diversity and ongoing evolution of GBS strongly support the need for current and future genomic monitoring among all 10 GBS serotypes since the information gleaned may have a high impact on the development of GBS vaccines.

In the clinical laboratory, WGS has proved valuable in hospital infection prevention programs by being able to identify and track outbreaks within a hospital [62,63]. A large portion of the published work has used WGS in tracking outbreaks of the most common nosocomial pathogens of methicillin-resistant *Staphylococcus aureus* [64,65,66] and *Clostridiodes difficile* [67,68,69]. WGS has aided in the tracking of outbreaks for the severe multi-drug resistant organisms such as carbapenem resistant *Klebsiella pneumoniae* [70,71,72], vancomycin resistant *Enterococcus faecium* [73,74,75], and multi-drug resistant *Acinetobacter baumannii* [76,77,78].

There are cases in the hospital setting where outbreaks are caused by more fastidious organisms, and WGS is used for identification and the epidemiologic tracking of these fastidious organisms, such as *Mycobacterium chimaera*. Back in the mid-2010s there was an increase in incidence of invasive *M. chimaera* infections in individuals with previous open chest cardiac surgery [79]. These *M. chimaera* isolates were sequenced and found to be genetically and epidemiologically linked to contamination of water heater-cooler units used in these patients’ cardiac surgeries [80,81,82]. These infections were not just an isolated incident as these water heater-cooler units were distributed world-wide [82,83,84,85]. In these published cases, WGS was performed in response to suspicion of an outbreak, but one can imagine that as technology becomes more efficient and cost effective that hospital infection programs can use these technologies via their diagnostic microbiology laboratories to help monitor and prevent future outbreaks.

As seen in the several examples mentioned above, WGS can not only provide identification and epidemiologic tracking of organisms but also give a full profile of the antimicrobial resistance genes present [86,87]. Current antimicrobial resistance detection methods are either culture-dependent phenotypic methods or culture-independent rapid molecular methods. The culture-dependent phenotypic methods are reliant on the growth of the organism to interpret potential resistance, and the culture-independent methods can only detect a few prominent resistance gene markers. The ability to provide a full genotypic antimicrobial resistance profile of an organism allows for a more comprehensive report on the potential antimicrobial resistance mechanisms present in an organism [86,87]. There are many published reports that show the promise of WGS for antimicrobial resistance prediction for conventional microorganisms such as *E. coli* [88,89], *S. aureus* [90,91], *Enterococcus faecium* [92,93], *Pseudomonas aeruginosa* [94,95], and *Neisseria gonorrhoeae* [96,97]. The major theme all these reports demonstrate is the ability of WGS genotypic resistance prediction to match the culture-dependent phenotypic resistance, which is imperative for even considering using WGS in place of the current methods. However, the current state of WGS technology does not allow for a complete replacement of current methods but rather an enhancement to them.

Currently, the turn-around-time is longer with WGS compared to the conventional culture-dependent and culture-independent methods for the conventional organisms mentioned above. The case where WGS can immediately make an impact for antimicrobial resistance prediction is for organisms that take a long time to grow or where antimicrobial susceptibility testing is laborious such as with *N. gonorrhoeae* previously cited and *Mycoplasma* and *Ureaplasma* species [98,99]. Another group of difficult to grow organisms where WGS can help accelerate the antimicrobial resistance detection is in slow growing Mycobacteria, such as multi-drug resistant *Mycobacterium tuberculosis* [100,101]. In one large consortium, known as the Comprehensive Resistance Prediction for Tuberculosis: An International Consortium (CRyPTIC), more than 10,000 isolates of *M. tuberculosis* were analyzed, and they found that the genotypic prediction for first-line tuberculosis drugs correlated with phenotypic susceptibility >90% [102]. A recent study confirmed that WGS can significantly decrease the turn-around-time it takes for traditional antimicrobial susceptibility, which can take up to a month to complete [103]. Another Mycobacterial antimicrobial resistance profile where WGS is helpful is with *Mycobacteroides abscessus* (formerly *Mycobacterium abscessus* [104]) which can have inducible resistance to clarithromycin [105], and the gold standard to detect this resistance is to incubate the broth microdilution for 14 days [106]. One group has validated and implemented a WGS test to predict this inducible resistance to clarithromycin as well as resistance to amikacin in a matter of 3 to 5 days compared to the standard of 14 days [107]. These cases of decreasing turn-around-time will facilitate providers in treating these difficult cases.

One caveat to antimicrobial resistance detection with WGS is that only known resistance genes and mutations are being detected. However, the continued sequencing of multi-drug resistant organisms can lead to the discovery of new resistance genes and resistance mechanisms. In fact, many groups are working to create machine learning software programs to look for antimicrobial resistance [108].

The use of WGS for fungal identification in clinical diagnostics is less established than for bacterial identification, but one can see where it may be more advantageous compared to the current morphological identification methods in mycology that may be subjective. One study showed the correlation is approximately 50% for phenotypic identification, using microscopic and colony morphology and physiologic studies, compared to sequencing of the D2 region of the large subunit of the rRNA gene and the full ITS regions of many common and uncommon clinically relevant molds [32]. WGS has been used in several outbreak investigations of contaminated medications such as in Chili and Colombia where anti-nausea medication was found to be contaminated with *Sarocladium kiliense*, which caused bloodstream infections in individuals [109]. Another example of fungal outbreaks due to medical contamination was with *Exserohilum rostratum* [110,111,112]. More than 13,000 patients were given various types of injections (epidural, paraspinal and joint) with contaminated methylprednisolone acetate (MPA) [110]. WGS was able to connect these cases and trace the contamination back to three lots of contaminated MPA.

One of the more notable instances of WGS aiding in fungal identification and epidemiological tracking is of the emerging multi-drug resistant yeast, *Candida auris*. *C. auris* has been reported to cause fatal infections and outbreaks in hospitals and long-term care facilities all over the world [113,114,115,116]. *C. auris* is known to be difficult to identify with standard laboratory methods due to its similar appearance and biochemical characteristics to other yeasts [117]. Conventional biochemical tests commonly misidentify *C. auris* as *Candida haemulonii* as well as with other yeasts such as *Candida parapsilosus* and *Rhodatorula spp.* [30,117]. There is an enrichment broth that can be used to overcome the misidentification of *C. auris*; however, it takes 21 days of incubation followed by an agar culture [118]. WGS was used to help confirm the identity of the organism and to provide epidemiology tracking of the organism all over the world [113,114,115,116,119].

Antifungal resistance prediction by WGS is being explored for yeasts such as *Candida* [120,121,122,123], but until recently no studies of mold antifungal resistance prediction by WGS had been published. However, multiple isolates of the ascomycete fungus *Aspergillus fumigatus* have now been investigated by WGS to study the dominant resistance mechanism in mutations of a gene that encodes a protein targeted by triazole antifungal drugs [124]. WGS was performed on a set of 24 isolates that were both azole-resistant and azole-susceptible from clinical and environmental sources from several countries [124]. A bioinformatics analysis of high-resolution SNPs confirmed the mutation of TR34/L98H as the sole mechanism of azole resistance [124]. Capitalizing on this approach, expanded studies of 218 *A. fumigatus* isolates, both clinical and environmental, from across the UK and Ireland were investigated by WGS to determine the molecular epidemiology of the fungus and to determine whether there was acquisition of drug-resistant isolates by at-risk groups. Data analysis confirmed that there were transmissions of azole resistant isolates from the environment. These data were further utilized to perform genome-wide association studies (GWAS) and pan-genome analyses to identify variations associated with itraconazole resistance, revealing potentially new and novel mechanisms of resistance with a polygenic basis [125]. The above examples illustrate the power of WGS in studying *Asperillus* and antifungal resistance, but there are numerous examples of clusters of clinically relevant fungi in the hospital and clinic settings where this technology can potentially unravel important epidemiological problems of acquisition and spread, as well as antifungal resistance. Work is needed to investigate and implement relevant WGS fungal identification into laboratories. Not only will identification and epidemiologic information be helpful, but also antifungal resistance prediction with WGS is a logical next step and could really improve treatment for fungal infections.

When it comes to implementation of NGS technologies in the clinical microbiology laboratory, the direct applicable NGS technology is WGS. WGS of a colony directly from a plate provides a wealth of knowledge that is comparable to the conventional microbiology workflow. However, WGS not only provides standard identification and antimicrobial resistance predication but also the ability to compare isolates to one another for outbreak detection. We believe implementation of WGS is a plausible initial step to bringing NGS into the laboratory because it complements and, in some cases, enhances the conventional microbiology laboratory workflow.

## 4. Direct Sample Metagenomics Sequencing

In thinking of the future of NGS in clinical microbiology, the implementation of metagenomics sequencing directly from a clinical sample offers the major advantage of eliminating the culture process entirely. This can drastically improve turnaround time and provide more timely answers to health care providers. There are two different NGS approaches of metagenomics sequencing that can be used to detect pathogens directly from a clinical sample (Figure 1). The first is an enrichment process known as deep amplicon or targeted sequencing where specific pathogen primers are applied to the extracted DNA to amplify out the desired group of pathogens (e.g., bacteria or fungi) or one specific pathogen of interest (e.g., HIV, SARS-CoV-2). The second is known as a shotgun metagenomics approach where all the extracted DNA or RNA from a clinical sample is sequenced, and the work to determine the potential pathogen is done in the bioinformatics pipeline. This approach allows for a wider net to be cast to look for potential pathogens of interest. Currently, there are still no Food and Drug Administration (FDA)-approved NGS tests for either of these approaches. Similar to the WGS tests in public health laboratories, there are laboratories certified by the Clinical Laboratory Improvement Amendments (CLIA) act that have laboratory developed tests (LDTs) in place for direct sample metagenomics sequencing. There are several commercial options for the shotgun metagenomics approach which will be discussed below.

### 4.1. Targeted Sequencing

Targeted sequencing is a method of sequencing where a selection or enrichment process is performed for an organism or a group of organisms of interest either prior to or after the library preparation process (Figure 1). There are several methods that can be used to target the specific organism or groups of organisms of interest, and they include: PCR amplification and probe hybridization. All these methods provide the advantage of less human DNA interference and a higher sensitivity of detection in sample types with large amounts of human cells (e.g., tissue or sputum). The primary disadvantage with these targeted sequencing approaches is the limited number of pathogens that one can detect. The performance of targeted sequencing directly from samples has been performed using both second and third generation technology platforms [126,127].

PCR amplification in targeted sequencing is also referred to as deep amplicon sequencing. Deep amplicon sequencing is an extension of PCR technology that offers a more in-depth coverage of the specific gene(s) of interest. The most well-known applications of deep amplicon sequencing are the amplification of 16S ribosomal RNA (16S rRNA) gene for bacterial identification [128] and 28S rRNA or ribosomal ITS genes for fungal identification [129]. Many laboratories have validated and implemented LDTs for deep amplicon sequencing with both bacterial and fungal identification [130,131], with one group even demonstrating a useful pipeline to incorporate both [127]. In addition, 16S deep amplicon sequencing has made it easier to identify the more difficult to grow organisms including tick-borne bacteria that usually are not detected with conventional bacterial culture (e.g., *Borrelia*, *Anaplasmsa*, *Ehrlichia*, and *Rickettsia*) [132]. Specifically, for 16S gene amplification, there has been clinical utility demonstrated for a variety of specimen types including joint fluid [133], blood [134], and cerebrospinal fluid (CSF) [135]. In the case of periprosthetic joint infections (PJI), conventional bacterial culture sensitivity is known to be imperfect due to patients receiving prior courses of antimicrobial therapy [136]. A comparison study was performed on patients with suspected PJIs to compare the sensitivity of targeted 16S sequencing to conventional bacterial culture to determine if there was an improvement in the identification of the organism causing infection [133]. A total of 47 PJI elbow joint fluids were positive by sequencing and four of them (8%) were negative by culture and positive with the targeted 16S sequencing [133]. There was a total of eight discrepant results between conventional bacterial culture and targeted 16S sequencing, and they were in PJI samples that were polymicrobial infections. In four of the cases, targeted 16S sequencing identified additional pathogens [133]. These data reflect the value and complexity of targeted 16S sequencing being able to provide additional information on these PJI, especially in the cases where they are culture negative. This study was performed retrospectively, but one can imagine that the additional information provided from targeted 16S sequencing could have changed treatment and patient outcomes.

The direct sample deep amplicon sequencing for fungal identification has been explored to a limited extent on fresh samples and has shown some success [32,137]. However, the one specimen type where deep amplicon fungal amplification has shown promise and great clinical utility is in formalin-fixed paraffin embedded (FFPE) tissues [137,138,139]. One of the conventional methods for detection of invasive fungal infections is the microscopic visualization of fungal elements in FFPE tissues coupled with a positive culture result [140]. Yet, there are many times when fungal elements will be detected in FFPE tissues, but there is a culture negative result or a culture was not ordered, since it may have been an incidental finding [137]. In addition, the fungal elements seen in FFPE tissue can be misidentified up to 21% of the time [141]. Deep amplicon sequencing for fungal organisms in FFPE tissues can offer providers an accurate identification of the organisms seen and allow them to employ the proper antifungal therapy option [137,138]. Specifically, ITS deep amplicon sequencing was shown to identify the fungal elements seen in FFPE tissue even when the slide had less than 50 fungal elements present [138].

The deep amplicon sequencing approach is used in clinical virology diagnostics, specifically with antiviral drug resistance in both cytomegalovirus (CMV) and human immunodeficiency virus (HIV). Both viruses have well known mutations in their genomes that confer resistance to specific antiviral drugs for each virus and are reviewed extensively elsewhere [142,143]. Commercially, the detection of the known antiviral drug resistance markers via sequencing for CMV is a LDT performed by both Viracor [144] and ARUP Laboratories [145]. Only Viracor offers an LDT of an antiviral drug resistance profile via sequencing for HIV-1 in addition to genotyping the HIV-1 integrase [146].

A popular targeted sequencing approach that is used in human genome sequencing is probe enrichment [147,148]. This method uses small hybridization probes ranging in length of base pairs and are assembled in panels of probes that can be as few as 50 probes up to millions of probes [149]. In the context of human genome sequencing and mutation panels, these hybridization probes are added to fragmented DNA and enrich for genes or mutations of interest [150]. This technology has been advanced for implementation in the clinical laboratory with the development of a bacterial probe hybridization panel known as a bacterial capture sequencing (BacCapSeq) system [151] and a viral probe hybridization panel known as Virome Capture Sequencing Platform for Vertebrate Viruses (VirCapSeq-VERT) [152]. There is no published work available to suggest that these specific probe hybridization panels are being used in the clinical microbiology diagnostic environment currently.

The probe hybridization method has been used in the public health setting to detect and track important global viral pathogens [153]. Specifically, with Zika virus, this method aided in lineage detection and epidemiologic tracking of the virus in the mid 2010’s outbreak tracking [154,155]. The success with Zika virus helped translate this approach to the current pandemic with the severe acute respiratory coronavirus 2 (SARS-CoV-2). Many public health laboratories have implemented a probe enrichment method of sequencing SARS-CoV-2 directly from specimens submitted to determine the variant that is circulating in the community. There is no consensus yet on whether there is a clinical need to genotype SARS-CoV-2 to the variant level [156], but the probe enrichment method does make this more of an attainable possibility if the laboratory has the capability.

### 4.2. Shotgun Metagenomics Sequencing

In contrast to targeted sequencing, shotgun metagenomes sequencing is a method used to cast a wider net since all the nucleic acid in a sample is sequenced (Figure 1). By sequencing all the nucleic acid, nearly all pathogens including bacteria, fungi, viruses, and parasites can be identified with one test [157]. This method of sequencing has been successful in detecting an infection from many different specimen types including sources that are normally sterile such as CSF [158,159,160,161], blood [162,163] and joint fluid [164,165]. In addition, it has been demonstrated to help in detecting an infectious agent in specimen types that have a documented microbiome present such as, respiratory tract specimens [166,167], gastrointestinal specimens [168] and urine [169]. One limitation to shotgun metagenomics sequencing is the background noise or interference of human nucleic acid or the resident microbiome, and this can be especially concerning in specimens such as tissues or respiratory secretions [158].

This approach has been implemented in several laboratories as an LDT, and these laboratories serve as a clinical reference testing center for patients. The first is at the CLIA–certified clinical microbiology laboratory at the University of California, San Francisco (UCSF) and they offer a shogun metagenomics sequencing test of CSF [158,159,170]. This test has an overall accuracy of 90% with a clinical sensitivity and specificity of 73% and 99%, respectively [158]. There have been various case reports that show the diagnostic utility of this test in cases where all other conventional diagnostic testing were negative including a case of neurocysticercosis from infection with *Taenia solium* [171], a case of West Nile virus [172] and a case of neurobrucellosis [173]. A one-year prospective multicenter clinical trial in patients presenting with a clinical picture of encephalitis, meningitis and myelitis was performed with this test to determine the usefulness of the test for the diagnosis of these illnesses [174]. The shotgun metagenomics CSF test identified more pathogens than the conventional direct detection testing of CSF (culture, antigen testing or rapid molecular methods) [174]. Yet, the authors did not make the claim that this test is at the stage to replace conventional diagnostic testing methods, but instead to enhance the diagnostic approach [174]. This same shotgun metagenomics sequencing pipeline has been applied to other body fluids including abscesses, joint fluid, peritoneal fluid, pleural fluid, bronchoalveolar lavage (BAL) and urine [127]. We must note that the application of this shotgun metagenomics sequencing pipeline on other body fluids is not available through this clinical laboratory reference testing center. The only shotgun metagenomics test offered is for CSF.

There are several commercial companies that have taken the methodology of shotgun metagenomics and created commercial assays for varying body sources. The first we will discuss is the Karius Blood/Plasma test. This test, referred to as a microbial cell-free DNA test or a non-invasive liquid biopsy, was first introduced into the infectious diseases diagnostics market in 2016. It is intended for non-invasive detection of deep-seeded and bloodstream infections. A single blood sample can detect >1250 targets [162,163] of diverse bacterial, fungal, DNA viruses and eukaryotic parasites. Specimens are frequently sent on complex patients with underlying risk factors for bacteremia, fungemia and invasive infection of various types, where routine cultures and complex molecular tests, such as, targeted 16S and 28S sequencing assays may be negative. The usual turn-around-time for a report is 24–48 h from time of receipt in the Karius laboratory in Redwood City, California [162]. The test is not FDA approved, but the Karius laboratory is certified under CLIA and is accredited by the College of American Pathologists for high complexity laboratory testing.

Because of its high frequency of use among general medicine physicians and infectious diseases physicians (both adult medicine and pediatric), this test will be described in detail here. Details of the sample preparation, sequencing (average of 45 million reads) and processing of sequencing output files and sequencing reads files have been described [163]. The analytical performance characterization [limit of detection (LOD) and limit of quantification (LOQ)] of this shotgun metagenomics sequencing assay has been described as well as the sensitivity, precision, and analytical specificity; clinical performance characterization was assessed by comparison with microbes identified by conventional testing [162].

In a patient report, the abundance for each detected target is expressed as DNA molecules per microliter (MPM). Results are reported graphically for detected targets so one can compare the number of times that target has been detected by Karius. In various studies, more than one target has been detected and reported [175]. The frequency of multiple pathogen detection is variable, as confirmed by the authors’ experience. All targets detected in a blood sample are reported hierarchically with the target with the highest abundance, MPM, listed first. One needs to assess the results and use clinical judgment to decide the clinical significance of some detected targets. The positivity rates (%) for detected pathogen(s) in samples submitted vary among age groups of patients and from institution to institution, but on average may be 50–70% [162,176,177]. With increased discrimination in ordering the test, the positivity rates may be more favorable.

Among the studies assessing the concordance of Karius positive sequencing results with conventional cultures is a study examining 2000 specimens tested by Karius where there was 97% agreement between positive sequencing results and blood cultures results in patients with sepsis [162]. There are several other diverse studies, some prospective and others retrospective, with relatively small numbers of patients, where concordance of Karius testing and culture or ribosomal sequencing ranged from 58–100% [178]. The two cases below exemplify the breadth of the test for non-cultivatable and cultivatable microorganisms and illustrate the utility of the Karius test in clinical infectious diseases. Some specific data and narrative were modified for anonymity of the patients.

CASE DESCRIPTION #1-A young child with DiGeorge Syndrome was 2 weeks postsurgical repair of a cardiac defect, still on a mechanical ventilator, and developed fever and bilateral pulmonary opacities. Blood and sputum cultures were non-revealing for bacteria and fungi. A blood specimen was sent for a Karius test and within 48 h the result was reported as 1365 DNA MPM of *Pneumocystis jirovecii*. The patient was started on specific therapy for *Pneumocystis jirovecii*, improved and was weaned off the respirator. A *P. jirovecii* PCR assay on an endotracheal specimen was positive also, several days later. Follow-up Karius test 2 weeks later was positive for *P. jirovecii* at 65 DNA MPM.

CASE DESCRIPTION #2- A child presented with a week of elevated fevers >103 F. On examination a previously undetected heart murmur of aortic regurgitation was detected, and an echocardiogram demonstrated a bicuspid aortic valve with a large vegetation and rupture of the valve. Originally, routine blood cultures were negative. A plasma Karius test was reported in 36 h as *Kingella kingae* with 1890 DNA MPM. Antibiotics were initiated and the patient had emergency aortic valve replacement. Follow-up Karius test 16 days later was positive for *Kingella kingae* at 55 DNA MPM. Aerobic blood cultures flagged positive at 9 days of incubation, and the same organism was recovered and identified by MALDI-TOF mass spectrometry and 16S rRNA sequencing.

More recent publications on the Karius test include a retrospective review of test utilization in a large Children’s Hospital in Texas, USA [175]. The findings concluded that, in general, conventional microbiologic testing provided the same results as the Karius test, but with shorter turn-around-time. In addition, no therapeutic management was different in the majority of patients when additional microbial agents were identified by the Karius test [175]. In contrast, a prospective study of this assay, compared to blood cultures and other standard microbiological testing, in adults with leukemia and febrile neutropenia revealed that the Karius results could have allowed earlier optimization of antimicrobials in 47% of patients [179]. This was a relatively small study of 55 patients, but with adjudication of clinical data, it was determined that infection was the cause of fever in 87% of the patients. In the opinion of many experts in the field of infectious diseases, the value of the Karius test is in the detection of unexpected, or difficult to detect or culture pathogens. An example was detection of *Rhizopus* in one patient in the above study at two different time points, where fungal culture and identification took several days [179].

Although follow-up Karius testing can be done on a patient, it is not usually repeated, and there is no controlled study demonstrating the merits of serial testing. In certain situations, such as a difficult to treat organism causing endocarditis or other unusual infections, one might invoke an argument for repeating the test to see the MPM decrease significantly after two to several weeks of antimicrobial therapy. Because the Karius test is so expensive ($2000 per test with added charges, at times significant, depending on the institution), some medical centers have stewardship processes in place (examples: consultation/vetting by local laboratory leaders in diagnostic microbiology and adult and pediatric infectious diseases) to assess the need for the test or any follow up Karius testing.

There are still many questions, unresolved, regarding the utilization and timing of ordering this test, the significance of polymicrobial results, and whether clinical outcomes mitigate the high cost of the test.

Another commercially developed shotgun metagenomics test is Explify™ Respiratory, which is performed primarily on BALs and was launched by IDbyDNA and ARUP Laboratories in 2017. Extraction of RNA and DNA from BAL specimens is followed by preparation of NG complementary DNA and DNA sequencing libraries and sequenced on NextSeq or NovaSeq instruments (Illumina) to a median depth of 3–5 million sequencing reads. Explify™ Respiratory can detect >900 bacterial, fungal, and viral respiratory pathogens [180]. Microorganism detection is based on detection thresholds, established by quality control studies, and results are reported semi-quantitatively. Results are also stratified and reported as potential pathogens or additional microorganisms based on known pathogenicity [180]. This clinical shotgun metagenomics NGS assay has been applied to the study of pneumonia in various patient groups, particularly in immunocompromised children and adults. These patients often have puzzling pneumonic processes that do not always yield answers from conventional bacterial and fungal cultures, nor from single or multiplex PCR assays. An example of its utility is in detecting missed pathogens in immunocompromised children with pneumonia. Previously missed putative microbial pathogens were identified in 18 of 41 (44%) of BALs in these children with life-threatening pneumonia, including 7 of 11 (64%) children with fatal infections. The Explify™ Respiratory assay detected a single pathogen in 12 children (63%), 2 in 5 (26%), and 4 pathogens in 1 (5%) patient. Bacterial (13), fungal (7) and viral (3) pathogens were detected in these numbers of children [181].

Similarly, 30 immunocompromised adults with 31 episodes of pneumonia underwent bronchoscopy and had BALs studied by the Explify™ Respiratory assay, and results were compared to conventional microbiologic testing (CMT). Final microbiologic diagnoses were seen in 11 cases (35%) with CMT alone, and in 18 cases (58%) with both CMT plus Explify™ Respiratory assay [180]. Final diagnoses were made in 20/31 cases (65%) by CMT only and in 23/31 cases (74%) based on CMT plus Explify™ Respiratory assay, not a great difference. The diagnostic advantage of the Explify™ Respiratory assay, however, was mostly the detection of additional bacterial causes, but appeared less useful, in this study for diagnosing fungal pneumonia [180]. It is apparent that clinical judgement should be applied to the interpretation of the Explify™ Respiratory results, as well as their significance in the context of patients’ clinical conditions. On occasion, there is a triumphant, but unexpected, result emanating from this Explify™ Respiratory assay; example, *Pneumocystis jiroveccii* (a yeast-like fungus) from an immunocompromised host.

Examples of the potential diagnostic and clinical value of the Explify™ test are two immunocompromised adult patients with pneumonia and negative conventional microbiologic testing who, separately, had only *Pantoea agglomerans* or *Ewingella americana* detected by the clinical metagenomics method performed on BALs [180]. Radiologic findings were consistent with pneumonic abnormalities. Although these are relatively uncommon organisms, they may have contributed to the pathology described in immunocompromised patients [180]. It is judicious to combine standard of care microbiology diagnostic approaches with this assay for diagnosis of puzzling pneumonic processes, especially in immunocompromised patients, to complement clinical decisions.

Finally, the commercial company IDbyDNA has expanded this metagenomics approach used in Explify™ Respiratory to the diagnosis of urinary tract infections with antimicrobial resistance markers, and several related presentations were delivered at the 32nd European Congress of Clinical Microbiology and Infectious Diseases, Lisbon, Portugal, 23–26 April 2022. This urinary pathogen ID/antimicrobial resistance Panel (UPIP) can detect >190 pathogens, including 135 bacteria, 35 viruses, 14 fungi, and 7 parasites and >2000 antimicrobial resistance markers [182]. Further details from peer-reviewed papers are anticipated to enlarge our understanding of the application and value of this metagenomics approach to urinary pathogen detection.

The application of shotgun metagenomics has come a long way in the various infections described above (i.e., meningitis/encephalitis, sepsis, pneumonia, and urinary tract infections). There are limited studies utilizing this approach to diagnose infections of bones, related tissues, and native or prosthetic joints. Data to evaluate the relative clinical value, impact on clinical outcome, or sensitivity and specificity are insufficient.

## 5. Factors to Consider When Implementing These Technologies

Regarding implementation within the laboratory, there are currently no FDA-approved tests for WGS but instead many laboratories certified with CLIA, including public health laboratories, have validated LDTs to perform the WGS including both the wet bench and bioinformatics workflows [183,184]. There are many factors to consider when trying to decide if introducing these sequencing technologies is the best for your laboratory. There are several excellent reviews that discuss this in more depth [185,186] but we will touch on a few of the main factors we consider to be the most important before implementing sequencing technologies in the clinical microbiology environment.

The first factor to consider is the laboratory validation process for these NGS applications (i.e., WGS, targeted metagenomics and shotgun metagenomics). Currently, there is no standardized kit or testing system to implement any of the NGS applications mentioned above that is FDA approved or cleared. This means that bringing on any of these NGS applications would require the laboratory to bring up the test as a LDT. The validation process for an LDT is very costly and time consuming, which limits the type of laboratories that would be able to adopt this type of technology. The parameters needed to validate an LDT can be difficult to meet with these NGS applications. The basic quality control aspects of an LDT for NGS are hard to determine since there no agreed upon reference standard in the clinical microbiology community. In the case of WGS, it is easy to sequence a positive type strain and a negative water control with each run to make sure the assay is running properly. However, with the metagenomics approaches, laboratories would have to assemble a mock community or spike sample with organisms of interest, and again, there is no agreement on the makeup of these mock communities or spiked samples. It is also difficult to find a gold standard method to compare accurately the results of the NGS application, since it has been shown in many cases that the NGS application is superior to the gold standard method in detecting microorganisms [186]. Recently, the CDC and the Association of Public Health Laboratories (APHL) created a web based reference that has the quality management systems tools and resources for laboratories to access when considering to validate these LDTs (https://www.cdc.gov/labquality/qms-tools-and-resources.html; accessed on 12 July 2022). However, even with these tools and resources available, the complexity of these NGS approaches and the rigorous validation process is currently keeping this technology in the space of reference laboratories and large academic medical centers.

The second factor to consider when implementing these NGS technologies is the reporting of the results, especially in the interpretation and clinical utility of the metagenomics approaches. The creation of bioinformatics pipelines to process the sequence data take considerable time and expertise, not only with the coding and computing side, but also the microbiological and clinical interpretation of the results. Currently, there are no commercialized bioinformatics pipelines to interpret the data; however, there are several large academic medical centers that have published their workflows for all three NGS applications of WGS [184], targeted sequencing [133] and shotgun metagenomics [174]. One aspect we favor, that several laboratories have recommended to help in the interpretation and reporting of the results from these NGS applications, is the creation and utilization of a group of individuals or an advisory board [174,184]. The members of the group/board would serve as a reference to help in the interpretation of interesting or inconclusive results and ensure that what was being reported concurs with the clinical picture.

A final major factor to consider when implementing these NGS approaches into the clinical microbiology laboratory is the cost. Although the cost to sequence a sample or organism has decreased, the cost to introduce this technology into the clinical microbiology laboratory space is great. One of the first obstacles with cost is to have the physical space to accommodate the wet lab portion of the test. In the wet lab workflow, it is important to have a laboratory that is adaptable to keep pre-amplification and post amplification material and spaces separate. This is to help reduce the possible contamination of the samples. Another major cost already mentioned was the validation of the LDT. In addition, once the test is validated and in place, it is hard to incorporate this test in the laboratory to replace the already low cost of conventional microbiology culture.

## 6. Future of NGS in Clinical Microbiology

NGS in the clinical microbiology laboratory is already a reality but only for a select few clinical microbiology laboratories with the budget and the personnel to make it possible. In this section we hypothesize and predict some of the potential future directions for this technology in diagnostic microbiology. First, the process of sequencing has dramatically decreased in price since the first generation of sequencing. It is conceivable that this technology with become cheaper allowing it to be more readily available to laboratories outside of large reference laboratories or large academic medical centers. We believe that sequencing could become inexpensive and have a fast enough turnaround time to the culture dependent methods. For example, WGS can be used to identify a colony growing on a plate and produce the AST profile of that organism in comparable times that it takes to run and get a simple identification with the conventional microbiological workup.

Considering AST testing, a major critique in implanting molecular AMR testing into the common microbiological workup is the concordance between phenotypic and genotypic AMR results. Even though an antibiotic resistance gene marker is detected in the genotypic profile, there is no guarantee that the gene is translated and transcribed to produce the enzyme or protein to confer resistance, unless seen in the phenotypic susceptibility results. In the future, it would be helpful to incorporate a proteomics workflow into the pipeline to see which proteins are being expressed to help infer phenotypic antibiotic susceptibility profiles [187].

Another area where significant progress can be made in the next 5 years is in the bioinformatics analysis, specifically for shotgun metagenomics sequencing. Current bioinformatics pipelines for shotgun metagenomics sequencing can detect the multiple pathogens that are present within a given sample. However, current technology does not allow for an assembly of a full genome of that pathogen unless it is a virus [174]. In the future, one can imagine that a bioinformatics pipeline for shotgun metagenomics of a clinical sample will be sophisticated enough to tease out true pathogen assembly of a complete genome with the ability to predict virulence and antimicrobial resistance markers.

## 7. Conclusions

NGS technologies have existed for several decades but are just beginning to change the diagnostic potential of clinical microbiology and public health laboratories. In these arenas, WGS allows for identification of fastidious organisms and for surveillance of potential outbreaks. In addition, WGS aids in the detection of not only known antimicrobial resistance genes and mechanisms, but also new genes or mechanisms that are not defined at this time. Targeted and metagenomics sequencing directly from samples helps increase the detection of organisms of interest, especially organisms where conventional methods are lacking in sensitivity or not available. These NGS technologies are changing the face of clinical microbiology diagnostics as we know it. While NGS cannot replace the conventional microbiology laboratory workup, the amount of information provided from sequencing will only enhance patient care.

## Data Availability

Not applicable.
